# Bayesian regression discontinuity designs: incorporating clinical knowledge in the causal analysis of primary care data

**DOI:** 10.1002/sim.6486

**Published:** 2015-03-24

**Authors:** Sara Geneletti, Aidan G. O'Keeffe, Linda D. Sharples, Sylvia Richardson, Gianluca Baio

**Affiliations:** ^1^ Department of StatisticsLondon School of Economics, LondonU.K.; ^2^ Department of Statistical ScienceUniversity College London, LondonU.K.; ^3^ Leeds Institute of Clinical Trials ResearchUniversity of Leeds, LeedsU.K.; ^4^ MRC Biostatistics UnitCambridgeU.K.

**Keywords:** regression discontinuity design, causal inference, local average treatment effect, informative priors

## Abstract

The regression discontinuity (RD) design is a quasi‐experimental design that estimates the causal effects of a treatment by exploiting naturally occurring treatment rules. It can be applied in any context where a particular treatment or intervention is administered according to a pre‐specified rule linked to a continuous variable. Such thresholds are common in primary care drug prescription where the RD design can be used to estimate the causal effect of medication in the general population. Such results can then be contrasted to those obtained from randomised controlled trials (RCTs) and inform prescription policy and guidelines based on a more realistic and less expensive context. In this paper, we focus on statins, a class of cholesterol‐lowering drugs, however, the methodology can be applied to many other drugs provided these are prescribed in accordance to pre‐determined guidelines. Current guidelines in the UK state that statins should be prescribed to patients with 10‐year cardiovascular disease risk scores in excess of 20%. If we consider patients whose risk scores are close to the 20% risk score threshold, we find that there is an element of random variation in both the risk score itself and its measurement. We can therefore consider the threshold as a randomising device that assigns statin prescription to individuals just above the threshold and withholds it from those just below. Thus, we are effectively replicating the conditions of an RCT in the area around the threshold, removing or at least mitigating confounding. We frame the RD design in the language of conditional independence, which clarifies the assumptions necessary to apply an RD design to data, and which makes the links with instrumental variables clear. We also have context‐specific knowledge about the expected sizes of the effects of statin prescription and are thus able to incorporate this into Bayesian models by formulating informative priors on our causal parameters. © 2015 The Authors. *Statistics in Medicine* Published by John Wiley & Sons Ltd.

## 1 Introduction

The regression discontinuity (RD) design is a quasi‐experimental design that estimates the causal effects of a treatment by exploiting naturally occurring treatment rules. Since its inception in the 1960's in educational economics 1, the RD design has successfully been applied in areas such as economics, politics and criminology 2, 3, 4, 5 amongst others. More recently, it has been reworked in the econometric causal inference literature 6, 7 and there has been some interest in the design in epidemiology 8, 9, 10, 11 and health economics 12.

The RD design can be applied in any context where a particular treatment or intervention is administered according to a pre‐specified rule linked to a continuous variable—referred to as the assignment variable. Such thresholds are common in many fields and, in particular, in primary care drug prescription. For instance, according to the National Institute for Health and Care Excellence (NICE) guidelines 13, statins (a class of cholesterol‐lowering drugs) should be prescribed in the UK to patients with 10‐year cardiovascular disease (CVD) risk scores in excess of 20%. Consider patients whose risk scores are close to the 20% risk score threshold; typically, there is an element of random variation in both the risk score itself and its measurement. Thus, we can consider the threshold to be a randomising device that assigns treatment (statin prescription) to individuals just above the threshold and withholds treatment from those just below the threshold. In other words, if we focus on an area close to the threshold, then we have a situation that is analogous to a randomised controlled trial (RCT), resulting in removal or mitigation of confounding where we can identify and estimate causal effects of treatments in primary care.

The RD design can be useful in situations where evidence from RCTs is available, as it is often the case that RCT results are not consistently replicated in primary care. In such situations, the RD design can shed light on why this might be the case. In other contexts, RD designs can confirm RCT results where other observational data might have failed to do so. Furthermore, RD methods, while not providing as substantive evidence of a causal effect as an RCT, are cheaper to implement, can be typically applied to much larger datasets and are not subject to as many ethical constraints. This could make such methods desirable in the overall accumulation of evidence regarding the effectiveness of a particular treatment, administered using strict prescription guidelines, on an outcome of interest in primary care. Finally, there are many situations where RCTs cannot be run, for example, in the case of experimental treatments for terminal diseases. The RD design means that doctors can administer the treatments to the severely ill but still obtain a valid (if local) causal effect of the treatment, provided they adhere to a strict guideline.

In this paper, our focus is two‐fold. Firstly, we formulate the RD design in the framework of conditional independence. This has, as yet, not been done, and we believe that it both clarifies the underlying assumptions and makes explicit the link with instrumental variables (IVs), of which the RD design is a special case.

Secondly, we introduce a Bayesian analysis of the RD design and illustrate its formulation, using an example on the prescription of statins in primary care. While Bayesian methods have been applied to the RD design, work has been principally on spline models 14, 15. We focus here on models incorporating prior information, which have not been widely considered, especially in primary care contexts. Because much is known already about the effect of statins on Low‐density lipoprotein (LDL) cholesterol, principally because of RCTs, we believe that this example is a good starting point for the application of Bayesian methods as strong prior information on the effect of statins is available. Furthermore, as part of the analysis, we are interested in estimating a causal effect for GPs who adhere to guidelines. This requires us to think carefully about formulating priors that are informative of the process that drives adherence. While the existence of robust information in this context facilitates the formulation of prior models, this is by no means a pre‐requisite of this methodology. We note that our principal motivation is not to replicate the results of RCTs or to solely estimate the causal effect of statins on LDL cholesterol using an RD design. Rather, we are interested in considering Bayesian methodology in an RD design and use the effect of statin prescription on LDL cholesterol as a motivating example.

We consider two applications of the methods, which are informative to a different degree, and examine how sensitive the results are to prior specification in datasets of different sizes. The discussion of the results highlights the importance of thinking carefully about prior specification and also that, in some contexts, it is not difficult to formulate plausible and realistic prior beliefs.

We use simulated data based closely on actual statin prescriptions in the health improvement network (THIN) primary care database to illustrate our Bayesian methodology and then apply this methodology to a subset of the THIN data.

The paper is organised in three parts: in the first one, Section 2, we first describe the RD design in more detail and introduce the running example (statins prescription for the primary care prevention of CVD). Then, we formalise the assumptions necessary to identify a causal treatment effect using the RD design. Finally, we clarify the links between the RD design and IVs and introduce the causal estimators.

The second part of the paper (Section 3) introduces the details of our novel Bayesian model formulation. In this section, we describe and justify all the distributional assumptions used in our model and discuss the implications of incorporating prior clinical knowledge in causal analyses, specifically when they are based on the RD design.

Finally, in the third part of the paper (Sections 4 and 5), we present the results of our analysis applied to a simulated dataset followed by a real data example. Problems and extensions are discussed in Section 6.

## 2 The regression discontinuity design

### 2.1 The basics of the regression discontinuity design

In its original inception, the RD design was used to evaluate the effect of schooling on a number of adult life outcomes, for example, income. The classic example considers scholarships that are offered to students according to their grade point average or other markers of academic/sporting ability. However, the RD design can be applied in any context where an intervention, be it a drug, a lifestyle modification, or other, is administered according to guidelines based on continuous variables.

These situations also arise commonly in primary care drug prescription: examples include the prescription of anti‐hypertensive drugs when systolic blood pressure > 140mmHg or of selective serotonin reuptake inhibitors for patients exhibiting more than four symptoms in the ICD‐10 classification of depression. Another interesting case, which we use as a running example in this paper, is the prescription of statins, a class of cholesterol‐lowering drugs, in the primary prevention of CVD, in the UK. There are clear NICE guidelines regarding statin prescription 13, which makes this a suitable case‐study to show the potential of the RD design to perform a causal analysis using primary care data. In the case of statins, the guidelines recommend that individuals who have not experienced a cardiovascular event should be treated if their risk of developing CVD in the subsequent 10years, as predicted by an appropriate risk calculator (e.g. Framingham risk calculator), exceeds 20%. Note that in the original NICE guideline, the choice of the threshold was driven also by cost‐effectiveness considerations.

A 10‐year cardiovascular risk score is predicted based on a logistic regression with a number of clinical and lifestyle factors. These typically include, amongst others, blood pressure, total cholesterol and smoking status. Thus the RD design can be used to estimate the effect of statins on clinical outcomes, specifically LDL cholesterol levels, in individuals around this threshold level.

#### 2.1.1 The sharp regression discontinuity design

**Figure 1:**
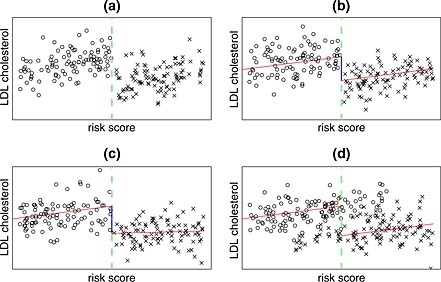
(a) The sharp regression discontinuity (RD) design with crosses indicating patients who have been prescribed statins and circles those who have not, (b) the sharp RD design with equal slopes with regression lines above and below the threshold and a bold vertical bar at the threshold to indicate the effect size, (c) the sharp design with different slopes and (d) the fuzzy design. Note that there are crosses below and circles above the threshold indicating that some general practitioners are not adhering to the treatment guidelines.

In an ideal situation, all general practitioners (GPs, UK family doctors) prescribe statins to patients who have a risk score above the 20% threshold and do not prescribe the drugs to those whose risk score falls below 20%. In addition, if statins also have a positive effect (i.e. they reduce LDL cholesterol), then a plot of risk score versus LDL cholesterol could look like Figure 1(a), particularly if cholesterol is linear in the risk score. Here, circles and crosses represent untreated and treated patients, respectively. The ‘jump’ at the 20% risk score can then be interpreted as the average treatment effect at the threshold. If we assume that the effect of statins is constant across risk scores, thats is, that the slope of the regression of LDL cholesterol against risk score is the same above and below the threshold, then the effect at the threshold can be considered an average effect for all risk scores, as in Figure 1(b).

It is possible however, that the slopes differ depending on whether the patient is above or below the threshold. In this case, the scatter plot of LDL cholesterol against risk score might look like Figure 1(c). In this situation, where thresholds are strictly adhered to, the RD design is termed *sharp*, and the value of the jump is estimated and interpreted as the causal effect at the threshold.

#### 2.1.2 The fuzzy regression discontinuity design

Typically in most applications, and particularly in the case of statin prescription, the RD design is not sharp. This is because GPs will often prescribe statins to patients below the threshold if they deem that it will be beneficial or possibly not prescribe statins to patients above the threshold in a subjective manner, rather than adhering to the threshold rule. We term this GP *adherence* to the guidelines. We contrast this to the situation where patients are not complying to the treatment prescribed. We make this distinction in order to avoid confusion by using the term compliance to describe the GP's behaviour when typically this term is used to describe patients' behaviour. For the remainder of the paper and, in particular, for the simulations, we assume that patients comply to their prescription. In Section 6, we briefly highlight differences between these two types of compliance and discuss how we might account for patient non‐compliance in the real data. When GPs do not adhere to treatment guidelines, a plot of risk score against cholesterol might look like Figure 1(d) where the crosses below the threshold and circles above the threshold indicate individuals who are not being prescribed according to the guidelines. In this situation, the RD design is termed *fuzzy*. In order to estimate treatment effects (typically local/complier effects), additional assumptions must be made as detailed in Section 2.2.

### 2.2 Assumptions

A number of assumptions must hold in order for the RD design to lead to the identification of causal effects. These assumptions are expressed in different ways depending on the discipline 6, 7, 16. We describe our approach in the language of conditional independence 17, 18, 19: in our view, this approach helps clarify situations where the RD design can be used and highlights the links with the theory of IVs. Throughout the paper, we follow standard notation: if a variable *A* is independent of another *B* conditional on a third *C*, then *p*(*A*,*B*∣*C*) = *p*(*A*∣*C*)*p*(*B*∣*C*) and we write *A*⊥⊥*B*∣*C* 18.

Let *X* be the assignment variable on which the treatment guidelines are based. Specifically, if *x*_0_ is the threshold given by the treatment guidelines, then let *Z* be the threshold indicator such that *Z* = 1 if $X\geq x_{0}$ and *Z* = 0 if *X* < *x*_0_. Furthermore, let *T* indicate the treatment administered (prescribed); we assume a binary treatment, so that *T* = 1 means treatment is administered (prescribed) and *T* = 0 means it is not. Also, let ***C***={***O*** ∪ ***U***} be the set of confounders, where ***O*** and ***U*** indicate fully observed and partially or fully unobserved variables, respectively. Finally, *Y* is the continuous outcome variable. In our case study, *X* is the 10‐year cardiovascular risk score with *x*_0_=0.2. Thus *Z* = 1 if a patient's 10‐year risk score exceeds 0.2 and *Z* = 0 if their score is below 0.2. The treatment is statin prescription (NB: patient taking the treatment). The outcome of interest is the level of LDL cholesterol.

We discuss in detail the assumptions necessary for the RD design in the following.

(A1) *Association of treatment with threshold indicator*:Treatment assignment must be associated with the treatment guidelines. This assumption can be expressed equivalently as
  Z⊥⊥?T,
  which implies that *Z* and *T* are not marginally independent. In the context of statin prescription this assumption will hold if the GPs adhere to the NICE treatment guidelines and *Z* is predictive of treatment *T*, *i.e*. they prescribe the treatment to patients with a 10 year risk score that exceeds 20% and do not prescribe statins to patients whose risk score is below 20%. This assumption can be tested directly by estimating the association between *Z* and *T*. This does not mean that the RD design breaks down when GPs prescribe according to their own criteria, as the guideline itself is still in place. What happens if some GPs prescribe according to their own criteria is that assumption A1 becomes weaker as the association between the threshold indicator (*i.e*. the guideline) and prescription practice decreases. However, provided the association is still strong, *i.e*. a sufficient number of GPs adhere to it, fuzzy methods can be brought to bear.
(A2) *Independence of guidelines*:The treatment guidelines cannot depend on any of the characteristics of the patient (excluding *X*), that is, they cannot be changed for individual patients. We can express this assumption in terms of the threshold indicator as
  Z⊥⊥C∣X,
  that is, *Z* is marginally independent of ***C***—and we note that this should hold at least around the threshold. We can also see this assumption as meaning that the patient characteristics (excluding *X*) cannot determine their value of *Z*.
  Assumption A2 does not preclude dynamic treatment strategies as long as these are pre‐specified. We could consider a dynamic strategy as one that depends not only on the risk score but also on a number of factors. For instance, a GP might look at a fixed number of (observed and recorded) risk factors when deciding whether to prescribe statins and only prescribe when a pre‐specified minimum number indicate elevated risk. This will be different for each patient but will not be different for two patients with the same values for the risk factors.
  If the threshold indicator is associated with some unobserved confounders ***U***, a weaker version of this assumption is that the threshold indicator does not depend on the unobserved confounders given the observed confounders ***O***
  Z⊥⊥U∣O.
  We can think of this as the RD design applied within strata of the observed confounders, for example, by considering statin prescription for men only.
  Neither version of A2 can be tested as each involves either implicitly or explicitly the unobserved confounders ***U***. However, A2 is likely to hold in one of the two forms, because it is typically externally imposed and does not vary from patient to patient or from one GP to another.
(A3) *Unconfoundedness*:In order for the RD design to be a valid randomisation device, the outcome must be independent of the threshold indicator, conditionally on the other variables. This can be expressed more formally as
  (1) Y⊥⊥Z∣(T,X,C).
  For the statin example, this requires that patients cannot determine their treatment assignment, that is, that even when they know about the treatment rule, they cannot manipulate their outcome in order to fall above or below the treatment threshold. This guarantees that there is some randomness in where subjects fall with respect to the threshold. While it is plausible for patients to try and persuade their GPs to prescribe statins when they do not have a high enough risk score, this is unlikely to happen in a systematic manner and can also be subsumed in a weakening of assumption A1. Nevertheless, equation (1) breaks down if the GPs systematically fail to adhere to the risk score guideline but rather base treatment decisions on unobserved factors. As total cholesterol is part of the risk factor and LDL cholesterol is in turn a part of the total cholesterol, it might appear that assumption A3 does not hold. However, total cholesterol also includes High‐density lipoprotein (HDL) cholesterol and the risk score contains a number of other factors. Thus the link between *Y* and *Z* in our example is not deterministic but subject to random variation generally and most importantly for individuals around the threshold; ***C*** will contain all the remaining confounders such as HDL cholesterol and thus there will be no direct link.
  The condition in equation (1) is also untestable as it too implicitly involves the unobserved confounders ***U***. It is therefore important to consider whether individuals on either side of the threshold really can be considered to be exchangeable.
(A4) *Continuity*:It is necessary to assume that, conditionally on the other variables, the expected outcome is continuous around the threshold *x*_0_. This can be expressed in terms of
  $$E(Y|Z,X=x,T,C)\text{is continuous in}x\;(\text{at}x_{0})\text{for}T=0,1.$$
  To understand why this assumption is necessary, note that the marginal expectation of the outcome, conditionally on the assignment variable alone, that is E(*Y*∣*X* = *x*), is in fact *discontinuous* around the threshold, and it is the size of the discontinuity that is interpreted as a causal effect. The continuity of the conditional expectation guarantees that it is the threshold indicator and not any of the other variables that is responsible for the discontinuity in the outcome. Some RD design texts 7 state this assumption in terms of the the limits from above and below of the expectation of *Y*. More generally, we can assume that the conditional distribution of *Y* given the two treatments (active and control) and the assignment are continuous at the threshold 6. This assumption is partly testable on the observed confounders ***O***, for example, if partial regression plots of the outcome against observed confounders conditional on the assignment exhibit discontinuities around the threshold, then assumption A4 is called into question.
  In the context of statin prescription, this assumption requires that the expected value of LDL cholesterol as a function of variables other than the risk be continuous. If there was a discontinuity in the association between LDL cholesterol and, for instance, body mass index (BMI) conditionally on the risk score being 20%, then it would not be possible to attribute the jump in LDL cholesterol to the threshold indicator and, as a consequence, the treatment. In particular, if BMI is a confounder for the relationship between risk score and LDL cholesterol, it would follow that the discontinuity observed in LDL cholesterol could be due to BMI.
(A5) *Monotonicity* (fuzzy design only):For the fuzzy design, another assumption is necessary in order to identify a *local* causal effect rather than an *average* effect (we formally define these in Section 2.3). This assumption requires that there are no GPs who systematically prescribe the opposite of what the guidelines recommend. We define the pair of prescription strategies that a GP has prior to seeing a patient as (*S*_*a*_,*S*_*b*_), for *a*bove and *b*elow the threshold, respectively. These are binary decision variables taking value 1 if the GP prescribes the treatment and 0 otherwise. Then we can express the monotonicity assumption as
  $$\mathrm{Pr}(S_{a}=0,S_{b}=1)=0,$$
  that is, the probability of there being GPs who would decide to prescribe the treatment to *all* individuals below the threshold and who would decide not to prescribe the treatment to individuals above the threshold is 0. We must also assume that the GPs act according to these prescription strategies. In the potential responses literature, this is often referred to as the ‘no defiers’ assumption. There are a number of weaker versions of the monotonicity assumption (for example, 20, 21), which are plausible in some RD design settings when the strong assumption given earlier cannot be assumed to hold.
  In the context of our running example, this seems a very plausible assumption: even if a GP is not in agreement with the guidelines, he or she will be concerned with patient benefit rather than in compliance with NICE recommendations. However, if we allow for patient non‐compliance to the treatment, then the monotonicity assumption implies that there are no patients who will, on principle, decide to do the opposite of what they are prescribed. It is likely that there are some of these patients in a real context and thus the weaker assumptions can be invoked. We discuss these briefly in Section 6. It is not generally possible to test this assumption unless we are able to inquire of GPs or patients how their decision strategy is formulated.

### 2.3 Links with instrumental variables and causal effect estimators

It is well known that the RD design threshold indicator *Z* is a special case of a *binary IV* 6, 19. We link the RD design to the IV framework using the language of conditional independence and thereby clarify how the RD design fits into the context of experiments and quasi‐experiments.

Consider the case of a binary treatment (e.g. an active drug treatment versus a placebo) and the two experimental designs commonly used for causal inference. The first is the ‘gold standard’, the double‐blinded RCT with perfect compliance, meaning that the individuals in the trial take exactly and only the treatment to which they have been assigned. The second is the RCT but with partial compliance (TPC), meaning that not all the individuals take the treatment they have been assigned.

In the RCT, it is possible to estimate the *average treatment (causal) effect*

(2) $$\begin{array}{cc} \text{ATE} & =E(Y|T=1)-E(Y|T=0)\\ =E(Y|Z=1)-E(Y|Z=0), \end{array}$$

without making additional assumptions, because randomisation and perfect compliance guarantee (bar unlucky and unlikely lack of balancing) that any difference in the outcome is due only to the treatment assigned.

In the TPC scenario, the average causal effect would be analogous to an ‘intention‐to‐treat’ estimator for the effect of the treatment on the outcome of interest, that is:

ITT=E(Y∣Z=1)−E(Y∣Z=0).

However, the ITT estimator would yield a biased estimate of the causal effect of treatment because there is confounding by treatment self‐administration. This means that some patients in the treatment arm (and we typically do not know which ones) have not actually taken the treatment or, conversely (and often less likely), that some of the patients in the control arm have obtained the treatment and taken it. Clearly, the threshold indicator (*Z*) alone does not represent a strict separation between the treated and the untreated, and we may not know what motivated the patients to act as they did, thereby introducing a bias into the estimation process.

To account for the fuzziness, and control for bias, we use a *local* (sometimes called a *complier*) *average treatment effect* (LATE) to estimate the causal effect of the treatment at the threshold. The LATE is defined as:

(3) $$\text{LATE}=\frac{E(Y|Z=1)-E(Y|Z=0)}{E(T|Z=1)-E(T|Z=0)}.$$

This estimator uses the threshold indicator as an IV for treatment, and it can be shown that the LATE yields an unbiased estimate of the treatment effect at the threshold, under the assumptions present in Section 2.2. We see that the LATE numerator is simply the ATE and that the LATE, in general, is a function of the ATE, scaled according to the difference in the probability of treatment above and below the threshold. The absolute value of this difference in probability of treatment will always be less than one, thereby implying that the LATE will always yield a causal effect estimate of a greater magnitude than the ATE (although not necessarily with the same sign). A difference in sign between the LATE and the ATE would imply that the probability of treatment above the threshold was less than that below the threshold, which would be highly unlikely under a valid RD design. The LATE is referred to as a *local* because it is only possible to estimate the treatment effect at the threshold for those patients who are able to take up the treatment given a change in the assignment variable at the threshold (i.e. the population of patients for whom E(*T*∣*Z* = 1) and E(*T*∣*Z* = 0) can be estimated).

In particular, it is necessary that the RD monotonicity assumption A5 holds. In words, this means that we assume that no GPs would prescribe treatment only to those patients whose assignment variables lie below the threshold and withold treatment only to those patients whose assignment variables lie above the threshold. If that was the case, then a proportion of the available data would comprise a ‘sharp’ RD design (for those GPs who prescribe according to the opposite of the threshold rule) and a ‘fuzzy’ RD design (consisting of data from those GPs who prescribe according to the threshold rule, albeit sometimes in a fuzzy manner). In essence, we would have a mixture of two RD designs in this situation, with opposite treatment effects, with respect to the threshold, and it is clear that an attempt to fit our RD design to such data would not result in an accurate or appropriate estimate of the causal effect at the threshold. However, in most situations, it is highly unlikely that there would be GPs who would always prescribe in a contrary manner to the treatment rule, and one would typically assume that no such GPs exist when attempting to fit an RD design. Nonetheless, this issue is an important one to consider for estimation purposes.

By comparing the RD design to the RCT and TPC scenarios described earlier, we see that the sharp RD design is analogous to the RCT and that the fuzzy RD design is analogous to the TPC with the treatment assignment corresponding to the threshold indicator. Thus, in a sharp RD design, the ATE is equivalent to equation (2), while for the case of the fuzzy design, where the threshold guidelines are not always adhered to, the LATE is a measure of the treatment effect at the threshold, with the threshold indicator as an IV.

This correspondence highlights the appropriateness of the ATE and LATE as causal effect estimates in the primary care context. The ATE is clearly the appropriate causal estimate for the sharp design as this is equivalent to the RCT. For the fuzzy design, the ATE as shown in equation (2) corresponds to the ITT estimator in a TPC. This ITT estimator is subject to confounding and does not identify a causal effect, and so the LATE is used to estimate the causal effect of the treatment at the threshold.

In our context, the LATE identifies the causal effect for those patients registered with GPs whose prescription strategy corresponds with NICE guidelines. We have no reason to believe that the types of patients registered with such GPs are systematically different to the patients of GPs whose strategies are different. Thus we believe that the LATE provides us with a valid and potentially generalisable causal effect estimate. A further discussion, involving lack of patient compliance to treatment is given in Section 6.

## 3 Bayesian model specification

Our motivation for using Bayesian methods to analyse data generated in a RD setting is three‐fold. Firstly, the Bayesian framework enables us to set priors in such a way as to reflect our beliefs about the parameters and potentially impose substantively meaningful constraints on their values. For example, given extensive RCT literature 22, it is widely accepted that the effect of statin treatment is a decrease in LDL cholesterol of approximately 2mmol/l. When modelling the LATE, we can parameterise the numerator (i.e. the sharp treatment effect ATE) in such a way as to express this belief, while still allowing for uncertainty around this informed prior estimate. We discuss strategies for achieving this goal in Section 3.2.

A second reason for adopting a Bayesian approach is that, when estimating the LATE, a major concern is that the denominator, that is, the difference between the probabilities of treatment above and below the threshold, can be very small at the threshold (i.e. when the threshold is a weak instrument). The Bayesian framework allows us to place prior distributions on the relevant parameters in such a way that the difference is ‘encouraged’ to exceed a certain minimum. This can stabilise the LATE estimate, as we discuss in Section 3.3.

Finally, standard frequentist methods rely on asymptotic arguments to estimate the variance associated with the treatment effect, which often results in overly conservative interval estimations. By contrast, Bayesian analyses are typically implemented using MCMC methods, which allow increased flexibility on the modelling structure, as well as relatively straightforward estimation for all the relevant quantities (either directly specified as the parameters of the model or derived using deterministic relationships amongst them).

The inclusion of (relatively) strong prior information makes sense especially in contexts where the signal in the data is particularly weak and confounded and when, as in the RD design context, information about both the drug treatment and the probability of treatment above and below the threshold is available through previous research and extensive content–matter knowledge. It is likely that such prior information could be obtained, either from observations in earlier datasets or pilot studies (perhaps relating to the probability of treatment above/below the threshold and/or hypothesised treatment effect sizes) or from elicitation through discussion with expert clinicians. However, in some cases, it is possible that little information might be known or hypothesised regarding prior beliefs about particular parameters of interest. This would not necessarily preclude the use of a Bayesian RD analysis, although the use of suitable vague prior distributions might be recommended.

It is also important to consider the effect of prior beliefs and choice of analysis method in the context of the RD design bandwidth. Clearly, the smaller the bandwidth, the smaller the number of data points included in an RD analysis. Using frequentist methods might be problematic because the standard errors of estimated parameters would naturally increase. However, as the bandwidth shrinks, we would expect the population of interest to become more homogeneous, under the assumptions presented in Section 2.2. In this case, it may be appropriate to hold reasonably strong prior beliefs regarding treatment effect, because the population for whom such beliefs would be held is likely to be fairly specific. This suggests that a Bayesian approach may be advantageous in such scenarios, although we note that the bandwidth should always be determined in a transparent and clinically relevant manner. Indeed, it would usually make sense to compare parameter estimates (Bayesian or frequentist) under a variety of different bandwidths, to check the sensitivity of results to bandwidth specification.

We discuss the strength of effect of the prior information when looking at the results of the analysis and the simulation studies, as well as to what extent results from these studies can be considered reliable in Section 4.

As the results appear to be more sensitive to priors on the denominator of the LATE, we summarise the priors for the ATE briefly in Section 3.2 before tackling the prior models on the denominator in more detail in Section 3.3.

### 3.1 Local linear regression

The estimators we consider depend on linearity assumptions, which do not always hold for the whole range of the threshold variable. This can put too much weight on data far from the threshold, thereby resulting in biased estimates. In this case, one possibility is to consider more flexible estimators, such as splines; this, however, is not recommended 23.

Alternatively, one can explore local linear regression estimators, which are obtained using data only within some fixed bandwidth, *h*, either side of the threshold. This achieves three aims: (i) to use the data around the threshold so that points further away have little or no influence on the predictions at the threshold; (ii) to make linearity assumptions more plausible, as a smaller range of points is used, which belong to an area where linearity is more likely to hold; and (iii) to obtain smooth estimates.

### 3.2 Models for the average treatment effect

In line with equation (2), we estimate the average LDL cholesterol level as a function of the threshold indicator. Firstly, we model the observed LDL cholesterol level separately for the individuals below (whom we indicate with *l* = *b*) and above (*l* = *a*) the threshold, as

$$y_{\mathrm{il}}∼\text{Normal}(\mu_{\mathrm{il}},\sigma^{2})$$

and specify a regression on the means

(4) $$\mu_{\mathrm{il}}=\beta_{0l}+\beta_{1l}x_{\mathrm{il}}^{c},$$

where $x_{\mathrm{il}}^{c}$ is the centred distance from the threshold *x*_0_ for the *i* ^th^ individual in group *l*.

Obviously, the observed value of $x_{\mathrm{il}}^{c}$ determines whether, under perfect GP adherence, the individual is given the treatment or not. Thus, for *l* = *a*,*b*, the expressions in equation (4) are equivalent to E(*Y*∣*Z* = 1) and E(*Y*∣*Z* = 0), respectively, and the ATE may be written

(5) $$\text{ATE}=\Delta_{\beta }=:\beta_{0a}-\beta_{0b},$$

that is the difference in the two averages at the threshold, that is, when $x_{\mathrm{il}}^{c}=0$.

Within the Bayesian approach, to complete the model specification, we also need to assign suitable prior distributions to the parameters (*β*_0*l*_,*β*_1*l*_,*σ* ^2^). Where possible, we use the information at our disposal to assign the values of the priors for the model parameters. For example, we know the plausible ranges of the risk score and the LDL cholesterol. We also know from previous studies, trials and conversations with clinicians, that LDL cholesterol increases with risk score and that once statins are taken, the LDL cholesterol tends to decrease. We attempt to encode this information in the priors later.

With (at least moderately) large datasets, the posterior inference is less sensitive to the distributional assumptions selected for the variance *σ* ^2^, because there is enough information from the observed data to inform its posterior distribution. As a result, we consider a relatively vague uniform prior on the standard deviation scale for the observed variable: *σ* ∼ Uniform(0,5). We note that this is extremely likely to be dominated by the information coming from the data and thus not particularly sensitive for the posterior distribution.

As for the coefficients for the regression models above and below the threshold, we consider the following specification:

(6) $$\beta_{0b}∼\text{Normal}(m_{0},s_{0}^{2})\;\text{and}\;\beta_{1b}∼\text{Normal}(m_{1b},s_{1b}^{2})$$

(7) $$\beta_{0a}=\beta_{0b}+\varphi \;\text{and}\;\beta_{1a}∼\text{Normal}(m_{1a},s_{1a}^{2}).$$

The priors on the parameters *β*_0*b*_ and *β*_1*l*_ for *l*∈{*a*,*b*} are chosen such that they result in LDL cholesterol levels that are plausible for the observed range of risk scores. This can be achieved by selecting suitable values for the hyper‐parameters $\left(m_{0},m_{1b},m_{1a},s_{0}^{2},s_{1b}^{2},s_{1a}^{2}\right)$ 1.

The parameter *φ* represents the difference between the intercepts at the threshold, that is, ‘jump’ due to the causal effect of the treatment. We consider two different specifications for *φ* upon varying the levels of informativeness on the prior distribution

$$\varphi^{\mathrm{wip}}∼\text{Normal}(0,2)\;\text{and}\;\varphi^{\mathrm{sip}}∼\text{Normal}(-2,1).$$

The former assumes that, on average, the treatment effect is null as the magnitude of the prior variance is in this case large enough that the data can overwhelm the null expectation and thus we identify it as weakly informative prior (*wip*). We indicate with the notation $\Delta_{\beta }^{\text{wip}}$ the ATE estimator expressed in the form of equation (5) resulting from this formulation of the priors.

In the latter, we encode information coming from previously observed evidence that statins tend to have an effect of around 2mmol/l at the threshold. In this particular case study, given the extensive body of RCTs on the effectiveness of statins, we set the variance to 1, which essentially implies relatively strong belief in this hypothesis. We term this the strongly informative prior (*sip*) and the resulting ATE estimator is $\Delta_{\beta }^{\text{sip}}$.

### 3.3 Models for the denominator of the local average treatment effect

Because we know that in clinical practice there is a clear possibility that the assignment to treatment does not strictly follow the guidelines, as there may be other factors affecting GPs decisions, we also construct a suitable model to compute the LATE estimator. To do so, we need to estimate the denominator of equation (3). We start by considering the total number of subjects treated on either side of the threshold, which we model for *l*∈{*a*,*b*} as

$$\overset{n_{l}}{\underset{i=1}{\sum }}t_{\mathrm{il}}∼\text{Binomial}(n_{l},\pi_{l}),$$

where *n*_*l*_ is the sample size in either group. The quantities *π*_*a*_ and *π*_*b*_ represent *E*(*T*∣*Z* = 1) and *E*(*T*∣*Z* = 0), respectively, and thus can be used to estimate the denominator of equation (3) as

(8) $$\Delta_{\pi }=:\pi_{a}-\pi_{b}.$$

As we have little information, *a priori*, on the probabilities of prescription above and below the threshold, we consider three different prior specifications for the parameters *π*_*l*_, leading to three possible versions of the denominator Δ_*π*_. We investigate the sensitivity of results to different beliefs regarding the strength of the threshold instrument by acting on the difference Δ_*π*_ directly. We give details here of an unconstrained and a flexible prior model. We have also formulated a constrained model with interesting properties albeit unreliable results. Additional details are presented in 24.

#### 3.3.1 Unconstrained prior for (*π*_*a*_,*π*_*b*_)

Firstly, we consider a simple structure, in which the probabilities on either side of the threshold are modelled using vague and independent prior specifications. For convenience, we use conjugate Beta distributions that are spread over the entire [0,1] range

$$\pi_{l}∼\text{Beta}(1,1).$$

Because this specification does not impose any real restriction on the estimation of the probabilities *π*_*l*_, we term this model unconstrained (*unc*), and we indicate the denominator resulting from the application of equation (8) under this prior as Δ*π* *unc*.

#### 3.3.2 Flexible difference prior for (*π*_*a*_,*π*_*b*_)

Finally, we construct a model in which prior information is used in order to ‘encourage’ a significant difference between the probabilities—we term this the flexible difference prior (*fdp*) and define it as

$$\text{logit}(\pi_{a})∼\text{Normal}(2,1)\;\text{and}\;\text{logit}(\pi_{b})∼\text{Normal}(-2,1).$$

**Figure 2:**
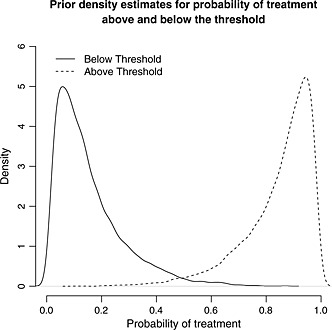
Prior predictive distribution for the probability of treatment below (solid line) and above (dashed line) induced by the flexible difference model. The former is substantially lower than the cut‐off value of 0.5, while the latter mostly exceeds this. Nevertheless, both allow for the full range of values in [0,1] to be possible.

These priors imply that, effectively, we assume the probability of treatment below the threshold to be substantially lower than 0.5 (i.e. most of the probability mass is concentrated in the range [0,0.5], while still allowing for a small chance of exceeding this interval). Similarly, we assume that most of the probability mass for *π*_*a*_ is above the cut‐off value of 0.5, as shown in Figure 2. In this way, we limit the possibility that the two quantities are the same, *a priori*, while not fixing a set difference between them. The denominator derived using these prior assumptions is indicated by Δ*π* *fdp*.

### 3.4 Models for the local average treatment effect

Suitable estimates for the LATE can be obtained by combining the models of Sections 3.2 and 3.3. We tried a number of combinations of different specifications. Eventually, we chose two as they were representative of the results. In all cases, the numerator is given by Δ^*sip*^ as the results were not sensitive to changes in the ATE. We combined

- the flexible difference model in the denominator with the strongly informative prior in the numerator and term this the *flexible* model
  $${\text{LATE}}_{\text{flex}}=\frac{\mathrm{\Delta @}{@}_{\beta }^{\text{sip}}}{\mathrm{@@\Delta @}{@}_{\pi }^{\text{fdp}}};$$
-  and the unconstrained denominator with the strongly informative prior in the numerator and term this the *unconstrained* model
  $${\text{LATE}}_{\text{unct}}=\frac{\mathrm{@@\Delta @}{@}_{\beta }^{\text{sip}}}{\mathrm{@@\Delta @}{@}_{\pi }^{\text{unc}}}.$$

## 4 Simulated data

We consider the simulation of data for which an RD design would be appropriate. We are interested in testing our methodology on data that are as close as possible to the data on primary care prescriptions. One reason is that results based on realistic data, with all its idiosyncrasies and quirks, are potentially of more value than simulations based on pre‐specified regression models. Another reason is that these data retain the basic structure of the original data so that the ranges of the variables of interest, LDL cholesterol levels, risk scores and so on are, for the most part, within the true levels of these variables. This means that it makes sense to think about prior information for the simulated data in much the same way as one would for the real data as it is as noisy as the real data and retains its quirks—see 25 for examples of Bayesian methods for weak IVs, which use data simulated from the ground up.

Specifically, we base our simulation scheme on THIN dataset (www.thin-uk.com). The THIN database is one of the largest sources of primary care data in the United Kingdom and consists of routine, anonymised, patient data collected at over 500 GP practices. Broadly, the dataset is the representative of the general UK population and contains patient demographics and health records together with prescription and therapy records, recorded longitudinally. Our aim is to use the models presented in Section 3 to estimate a pre‐defined treatment effect of the prescription of statins on LDL cholesterol level (mmol/l). We base our simulation scheme on a subset of data from THIN consisting of men only aged over 50years (*N* = 5720 records). The simulation study is described in detail in the supplementary material.

We aim at examining the properties of the estimators presented in Section 3 under varying levels of unobserved confounding and instrument strength (i.e. how strongly the threshold is associated with the prescription). For the sake of simplicity, we considered the HDL cholesterol level because it is predictive of both LDL cholesterol and treatment as the only unobserved confounder. The estimated correlation between the LDL and HDL cholesterol levels is 0.18 in the original dataset that was used used as a basis for the simulated data. To increase the level of unobserved confounding, we also use an adjusted dataset in the simulation, in which the estimated correlation between the LDL and HDL cholesterol levels is increased to 0.5. Overall, we define four levels of unobserved confounding where unobserved confounding increases with level number. We also consider cases in which the threshold acts as either a *strong* or a *weak* IV for the treatment. This is achieved through the pre‐defined choice of a regression parameter during the simulation algorithm. Details of both are provided in supplementary materials.

### 4.1 Simulation–results

**Figure 3:**
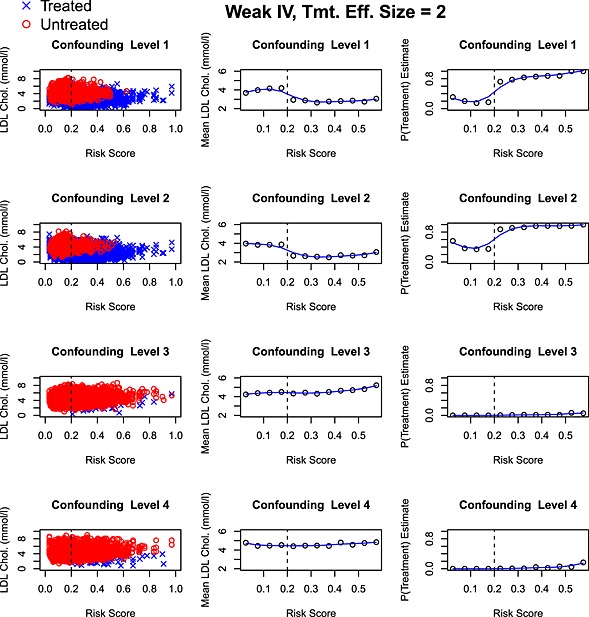
Plots in the left hand column show risk versus simulated LDL cholesterol level, those in the central column show risk score (bin mid‐point) versus sample mean LDL cholesterol level and those in the right‐hand column show risk score (bin‐midpoint) versus estimated probability of treatment. Plots are shown for different levels of confounding using simulated datasets with a treatment effect of size 2 and threshold acting as a strong instrument for treatment. A dashed vertical line indicates the threshold level.

We simulated 100 datasets using the algorithm described in Section 4 and fitted models using each of them. It is often not clear whether or not there exists a discontinuity at particular threshold, especially when data are very variable. We investigated this further by producing plots of the raw data points (continuous threshold variable against outcome) and by producing plots showing outcome mean estimate and raw probability of treatment estimate within regular bin widths defined by the threshold variable (in this case, the risk score). This is a common initial exploratory analysis when an RD design is thought to be appropriate and is typically used as a tool to back up the assumptions, which determine whether or not an RD design is valid 4, 6, 16, 26. Figure 3 shows such plots produced using one of the simulated datasets described earlier, under each defined level of unobserved confounding for a strong instrument using the design threshold. In each case, the treatment effect size is 2. A similar plot produced using datasets where the threshold is a weak instrument for treatment, which can be found in Figure 1 in the supplementary material. The raw plots (left‐hand column) show clearly that the RD design becomes more fuzzy as confounding increases, especially where the threshold is a weak instrument for treatment. The plots of the mean outcomes (central column) and estimated probabilities of treatment (right‐hand column) show obvious discontinuities at the threshold value of 0.2. The discontinuities are generally larger for lower levels of unobserved confounding. Splines are added to these plots to highlight the underlying pattern. When plots of either the estimated outcome means or raw estimates of probability of treatment—within risk score bins—exhibit a jump in at the threshold, then there is evidence to suggest the use of the RD design is appropriate. In light of these initial plots, an attempt to implement the RD design appears reasonable in all scenarios except where threshold is a weak instrument for treatment and unobserved confounding is at a high level. We performed analyses using RD designs on each of the 100 simulated datasets for all levels of unobserved confounding and instrument strengths for threshold. Results were combined for each unobserved confounding/instrument strength level, and we now present some of these results. As we are operating within the local linear regression framework, we considered three bandwidths (0.05, 0.15 and 0.20) within which to perform the linear regressions.

We found that, across all considered bandwidths and treatment effects, data at confounding levels 1 and 2 showed similar results and, in addition, data at confounding levels 3 and 4 showed similar results for both instrument strengths. This is perhaps not too surprising because the only difference between these scenarios is in the estimated correlation between LDL cholesterol level and HDL cholesterol level. Hence, for brevity, we present tables of results that only include unobserved confounding levels 1 (low level of unobserved confounding) and 3 (high level of unobserved confounding). Furthermore, we show results for a simulated treatment effect of size 2 and for chosen bandwidths 0.05 and 0.25. The bandwidth of 0.15 and treatment effect sizes of 0.5 and 1.09 were also considered, full results are available on request from the authors.

Tables 1 and 2 show results from the simulation studies with treatment effect set to 2 (i.e. treatment with statins is associated with a reduction of 2mmol/l) for chosen bandwidths 0.05 and 0.25, respectively. For frequentist estimators, parameter estimates and associated standard 95% confidence intervals were calculated by combining estimates from simulations using Rubin's rules. For Bayesian estimators, sample means of the posterior means and of the 95% credible interval limits from simulations are reported. We include results using ATE estimators obtained by estimating the regression model (4) using a standard frequentist analysis, which we term ${\mathrm{@@\Delta}}_{\beta }^{\text{freq}}$, along with all Bayesian estimators described in Section 3.

**Table 1:** Simulation study results over 100 simulated datasets, for various confounding scenarios and instrument strengths for threshold.

*Bandwidth = 0.05, Treatment Effect Size = 2*						
		ATE estimators			LATE estimators	
IV	Confounding	Δ*β* *freq*	Δ*β* *wip*	Δ*β* *sip*	LATE_unct_	LATE_flex_
Strong	1: Low	−1.74	−1.86	−1.87	−2.10	−2.10
		(−1.98, −1.51)	(−1.98, −1.74)	(−1.99, −1.74)	(−2.25, −1.95)	(−2.26, −1.96)
	3: High	−0.74	−0.89	−0.90	−2.20	−2.20
		(−1.08, −0.41)	(−1.02, −0.76)	(−1.03, −0.76)	(−2.59, −1.83)	(−2.59, −1.83)
Weak	1: Low	−1.01	−1.16	−1.17	−2.19	−2.18
		(−1.31, −0.72)	(−1.29, −1.03)	(−1.30, −1.04)	(−2.49, −1.91)	(−2.48, −1.90)
	3: High	0.05	−0.08	−0.09	−45.72	−15.75
		(−0.16, 0.25)	(−0.20, 0.04)	(−0.21, 0.03)	(−311.52, 207.84)	(−87.39, 29.38)

ATE, average treatment effect; LATE, local average treatment effect.

**Table 2:** Simulation study results over 100 simulated datasets, for various confounding scenarios and instrument strengths for threshold.

*Bandwidth = 0.25, Treatment Effect Size = 2*						
		ATE estimators			LATE estimators	
IV	Confounding	Δ*β* *freq*	Δ*β* *wip*	Δ*β* *sip*	LATE_unct_	LATE_flex_
Strong	1: Low	−2.02	−1.98	−1.98	−2.26	−2.26
		(−2.17, −1.87)	(−2.08, −1.88)	(−2.08, −1.89)	(−2.38, −2.14)	(−2.38, −2.14)
	3: High	−0.97	−0.94	−0.94	−1.90	−1.90
		(−1.27, −0.67)	(−1.04, −0.83)	(−1.05, −0.84)	(−2.14, −1.66)	(−2.14, −1.66)
Weak	1: Low	−1.25	−1.24	−1.25	−2.11	−2.10
		(−1.47, −1.04)	(−1.35, −1.14)	(−1.35, −1.14)	(−2.31, −1.91)	(−2.31, −1.91)
	3: High	−0.20	−0.18	−0.19	−25.28	−22.85
		(−0.31, −0.08)	(−0.27, −0.08)	(−0.28, −0.09)	(−49.48, −10.15)	(−48.68, −9.12)

ATE, average treatment effect; LATE, local average treatment effect.

Examining Tables 1 and 2, we see that the Bayesian LATE estimators generally capture the true value of the treatment effect (‐2.00) and provide plausible 95% credible intervals for both confounding levels where threshold is a strong instrument for treatment and for the low unobserved confounding level where threshold is a weak instrument for treatment. In general, both Bayesian and non‐Bayesian ATE estimators do not reflect the true treatment effect, especially as the unobserved confounding level increases and the strength of threshold as an instrument weakens. An exception is when the bandwidth is large (0.25), the level of unobserved confounding is low and the threshold is a strong instrument for treatment. This may be expected as the RD design might be considered almost sharp under these conditions. In addition, a relatively large bandwidth of 0.25 ensures that there are many treated individuals above the threshold and many untreated individuals below the threshold and, in such cases, an ATE estimator may be considered appropriate. The larger amount of utilised data for the bandwidth of 0.25 may also explain why the frequentist ATE estimates are more similar to the Bayesian ATE estimates in Table 2 when compared with those in Table 1. In general, there is some bias in most estimates, possibly as a result of different sources of noise incorporated into the simulation set‐up, together with unobserved confounding and changing instrument strength.

Where unobserved confounding is high and the threshold is a weak instrument for treatment, we see that all estimators behave in an unpredictable manner and fail to estimate the treatment effect accurately. This is not surprising because the design becomes too fuzzy for the modelling techniques presented to be applicable. Refer to Figure 1 in the supplementary materials for a visual confirmation. Similar problems are seen in simulation studies investigating the effect of weak instruments with unobserved confounding 25.

We considered a number of prior specifications in this work. In situations where such information was available, for example, the possible size and nature of the effect of statins on LDL cholesterol levels based on clinical trial results and/or expert GP knowledge, we attempted to account for this. Where less information was available, as in the case of the probabilities in the denominator for the LATE, we attempted to understand the sensitivity of results to prior specification.

Overall, the effect of the prior information appears to be negligible for the ATE, with the Δ*β* *wip* and Δ*β* *sip* ATE estimators producing similar estimates across all scenarios and for both bandwidths. Similarly, there are no obvious differences between the LATE_unct_ and LATE_flex_ estimators under these different prior distributional assumptions. We would generally recommend using the flexible prior models as they do provide some stability when the denominator of the LATE is very small. In the next section, we consider an application of these methods to a set of real data on statin prescriptions in UK primary care.

## 5 Example: prescription of statins in UK primary care

In this example, we considered a subset of patients from THIN (which we described in Section 4). The THIN scheme for obtaining and providing anonymous patient data to researchers was approved by the National Health Service South–East Multicentre Research Ethics Committee in 2002. Approval for this study was obtained from the Scientific Review Committee in August 2014. We used data from male patients aged 50–70 who were non‐diabetic, non‐smokers, had not previously received a statin prescription nor experienced a CVD event and for whom 10‐year CVD risk score was recorded by the GP during the time between 1 January 2007 and 31 December 2008; there were 1386 such patients. The selection of this group is consistent with NICE guidelines, stating that statin therapy should be initiated in individuals whose 10‐year risk of experiencing a CVD event is greater than 20% in the under 75s, which were released in January 2006. Using data from 2007–2008 allows time for the policy to be adopted by UK GPs.

The intervention is the first prescription of statin therapy, and the outcome variable is the LDL cholesterol level (mmol/l), where LDL cholesterol level is recorded between 1 and 12months after the calculation of the risk score. Of the 1386 patients in our data, 705 (50.9%) initiated statins during the period considered. We note here that the subset of patients in this example is fairly restricted, and consequently, any results we report are not representative of the general population or even subgroups of clinical interest.

### 5.1 Example–results

**Figure 4:**
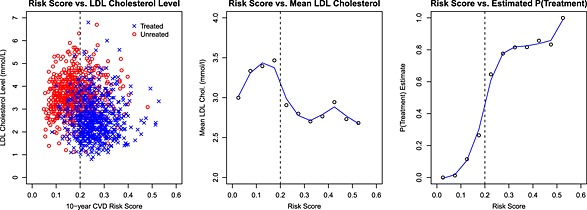
The left‐hand plot shows 10‐year CVD risk score versus LDL cholesterol level, the plot in the centre shows risk score (bin mid‐point) versus sample mean LDL cholesterol level and the plot in the right‐hand column shows risk score (bin‐midpoint) versus the estimated probability of the treatment. A dashed vertical line indicates the threshold level of 20%.

Following the simulation study in Section 4, we considered firstly appropriate plots to determine whether or not a RD design was suitable for these data. Figure 4 shows three plots in a similar manner to Figure 3. The centre and right‐hand plots indicate obvious discontinuities in the LDL cholesterol level in the probability of prescription at the threshold. The raw (left‐hand) plot also indicates that there is fuzziness present in the data. Overall, these plots suggest that an RD is appropriate and that, due to the fuzziness, LATE estimators should be considered as more reliable effect estimates than their ATE counterparts.

Next, we fitted the models described in Section 3 to produce a table of results analogous to those shown in Tables 1 and 2. Table 3 shows the estimates obtained when fitting our models to these real data; as before, two bandwidths of 0.05 and 0.25 were considered. We note that, unlike our simulation study, we do not know the true treatment effect of statins.

**Table 3:** Table of treatment effect estimates from an regression discontinuity design fitted to a subset of The Health Improvement Network data. Intervals are 95% credible intervals or, for non‐Bayesian estimates, 95% confidence intervals.

	ATE estimators			LATE estimators	
Bandwidth	Δ*β* *freq*	Δ*β* *wip*	Δ*β* *sip*	LATE_unct_	LATE_flex_
0.05	−0.29	−0.53	−0.55	−1.44	−1.41
	(−0.58, −0.01)	(−0.73, −0.40)	−(0.69, −0.40)	(−1.96, −0.97)	(−1.92, −0.96)
0.25	−0.54	−0.54	−0.53	−1.02	−1.00
	(−0.71, −0.37)	(−0.68, −0.41)	(−0.70, −0.39)	(−1.31, −0.74)	(−1.31, −0.70)

ATE, average treatment effect; LATE, local average treatment effect.

Examining Table 3, we see that, in general, the LATE estimates appear to capture a treatment effect for both bandwidths. Both flexible and unconstrained Bayesian LATE estimators (LATE_flex_ and LATE_tunct_) produced similar estimates of the treatment effect (ranging from −1.00 to −1.44) for both bandwidths. All 95% confidence and credible intervals for the LATEs indicated a significant departure from zero, suggesting that the initiation of statin therapy may cause a reduction in LDL cholesterol level for this subset of patients. In general, the Bayesian ATE estimates were similar for each bandwidth and tended to be closer to zero than those using the Bayesian LATE estimators (with estimates ranging only from −0.55 to −0.53 for Bayesian ATEs across both bandwidths). We note that we would always expect the ATEs (Bayesian or frequentist) to be smaller than the corresponding LATEs, owing to the construction of the LATE (equation (3)). However, with such a discrepancy in magnitude between the ATE and LATE estimates, and the obvious fuzziness in the data, it is likely that, in this particular case, the LATE represents a more accurate estimator for the treatment effect at the threshold.

For the smaller bandwidth of 0.05, the frequentist ATE estimate of −0.29 was closer to zero than any of the Bayesian estimators, although results were close for the larger 0.25 bandwidth. The difference in the frequentist ATE estimates is probably due to the inclusion low risk individuals who have lower LDL cholesterol in the analysis based on the larger bandwidth. These are represented by the point on the far left in the middle plot of Figure 4. The frequentist regression below the threshold becomes flatter, and the intercept decreases leading to a smaller effect estimate.

The difference in the LATE estimates using different bandwidths is also due to the inclusion low risk individuals, however it is the denominator that is affected as the Bayesian ATEs are robust to changes in bandwidth. When the larger bandwidth is used, it leads to the inclusion of individuals who have a close‐to‐zero probability of being treated (because they are low risk) below the threshold and the inclusion of individuals who have a close to one probability of being treated (because they are high risk) above the threshold. These are the points to the far left and far right of the right hand plot in Figure 4. As the denominator of the LATE is the difference in the probability of treatment above and below the threshold, it increases in value.

The further individuals are from the threshold the more likely it is that including them in the analysis will violate the RD assumptions. However, using a larger bandwidth typically means a larger sample of individuals and hence more power for the analysis. In this example the sample sizes range from 680 to 1377 for bandwidths 0.05 to 0.25. The estimates based on the smaller bandwidth have sufficient power, and RD assumptions are less likely to be violated.

## 6 Discussion

### 6.1 Critical issues

#### 6.1.1 ‘Local’ versus ‘global’ effect

An apparent drawback of the RD design is the ‘local’ nature of the causal estimate, that is, there is no guarantee that the causal effect is the same over the whole range of the risk score. If the aim of estimating the causal effect is to compare it with the results of trials and to determine whether the prescription guidelines are effective, the local nature is not a disadvantage. Rather, it will highlight whether the guidelines need to change if the results are starkly different from those of a (well‐conducted) trial. Furthermore, while trials may indicate that the effect of statins is constant across strata of age, sex and initial cholesterol levels, there is no reason to assume that this applies across risk scores in the general population treated by GPs, especially when partial compliance of patients to prescriptions is to be expected. In Section 6.2, we discuss how multiple thresholds might be used to determine whether the effect is constant across the range of the assignment variable.

#### 6.1.2 Sensitivity of results to choice of bandwidths

As highlighted in the example in Section 5, there is inherent in the RD design, a tension between using points within a small bandwidth of the threshold so that the RD assumptions hold and using larger bandwidths to improve reliability of estimates. Results can be sensitive to large changes in bandwidth especially in situations where the design is very fuzzy as seen in the simulation study in Section 4. There are some recommendations in the literature regarding the optimal size of a bandwidth 6 however these appear somewhat arbitrary. We suggest that researchers with context‐specific knowledge decide on an appropriate bandwidth such that the RD assumptions can be assumed to hold but sufficient data are available to obtain reliable estimates of parameters of interest. This process will generally include a sensitivity analysis.

#### 6.1.3 Compliance and adherence

In the context of the case study on which our simulations are based, we have two types of ‘compliance’. One is the adherence of the GP to the prescription guidelines, which we have assumed to be partial, in our simulations. The second is the compliance of the patient to the treatment prescription, which in contrast we have assumed is perfect. In real data, this is hardly ever the case: many patients do not take statins when they have been prescribed.

This aspect also relates to the fact that the LATE estimates a causal effect of a treatment in a population defined by the fact that the GP adhered to the prescription guidelines. We can ask two questions here. Firstly: are patients whose GPs adhere to guidelines comparable to those whose GPs have alternative strategies? Secondly: given that we are interested in comparing the RD design results from primary care to those of RCTs, are RCT participants comparable to patients whose GPs adhere to guidelines?

The first question means we need to understand whether GPs who prescribe according to the guidelines have patients that are systematically different from those who have GPs with alternative treatment strategies. There might be circumstances in which this is the case, for example, if different primary care trusts have different treatment ‘cultures’ as well as different patient populations. To answer the second question, we must consider that individuals recruited into an RCT are often selected on the basis of characteristics that make them more likely to comply with, and respond to, treatment and that a primary care population will not necessarily be similar in those respects. Such scenarios should be considered carefully when considering the use of an RD design in a primary care setting.

### 6.2 Future work

Problems with GP and patient compliance result in the potential invalidity of Assumption 5, which is necessary to identify the LATE. This assumption states that there are no GPs whose prescription strategy is to refuse to adhere to the guidelines.

This would suggest that GPs have treatment strategies in place before seeing patients and that they act according to these strategies. While this may be plausible for GPs, it is unlikely to apply to patient compliance. In this case, we would be inferring that patients have strategies regarding compliance to taking medication in place before they are prescribed and that they act in accordance to these strategies. Moreover, we would also have to assume that there are no patients whose strategy it is to ‘defy’ the prescription. Both aspects of the assumption are less credible as patients are less likely to have strategies, and there are likely to be patients who will try to do the opposite of what they are ‘told’. We mention this here, in order to support our use of the LATE and to distinguish it from the more common situation of patient compliance where it is used and potentially less reliable. In dealing with patient compliance, we recommend limiting the RD design to those patients whom we consider exchangeable, so that we may not need to introduce additional complexity within the models to account for patient non‐compliance. Further work in this respect is required but is outside the scope of this paper.

Our focus has been here on statin prescription, where strong information can be brought to bear in prior model formulation. With other treatments and outcomes, it may be that there is limited knowledge regarding the effect of the treatment on the outcome (generally confined to a specific sub‐population of patients) or of clinical adherence to treatment guidelines, but that there may be a large amount of real observational data from primary care. We believe that it would be useful to apply Bayesian RD methods in such a scenario to combine limited evidence‐based and clinical prior beliefs with actual observed data in an effort to assess treatment effects in clinical practice and perhaps inform whether or not further trials/experiments should be considered.

We believe that the RD design has a great potential in primary care. Given the move towards pragmatism in clinical trial design, and the use of routine electronic health databases for patient follow up in trials, future trial results are likely to be augmented by planned RD designs, with thresholds at different levels of the assignment variable, in order to determine where in disease progression the treatment is most effective in primary care as well as having a more realistic basis for cost‐effectiveness analyses. This is particularly relevant when the treatment targets individuals who are likely to be under‐represented in trials, or when the treatment is for specific subgroups of the population, such as patients who are terminally ill or who suffer from rare diseases. Additional model assumptions or adjustments may be required when fitting an RD design to such subgroups.

## Supporting information

Supporting info item

## Footnotes

1
^1^For instance, selecting *m*_0_=3.7, *m*_1*b*_=8, *s*_0_=0.5 and *s*_1*b*_=0.75 implies that the prior 95% credible interval for the estimated LDL level ranges in [2.57;4.83] mmol/l for individuals with a risk score of 0 and in [2.72;4.68] mmol/l for individuals close to the threshold. For the slope *β*_1*a*_ above the threshold, we encode the assumption that the treatment effect is subject to a form of ‘plateau’, whereby for individuals with very high risk score, the effect is marginally lower than for those closer to the threshold. See the online supplementary material for details.
