# Supplementary material for: Bayesian regression discontinuity designs: incorporating clinical knowledge in the causal analysis of primary care data
Source: Stat Med. 2015 Mar 24;34(15):2334–52. doi: 10.1002/sim.6486 (PMC4856212; doi:10.1002/sim.6486)
Supplement: Supplementary file 1 — Supporting info item [file SIM-34-2334-s001.pdf]

Research Article

Statistics in Medicine

Received XXXX

(www.interscience.wiley.com) DOI: 10.1002/sim.0000

Supplementary Material for Bayesian regression discontinuity designs: Incorporating clinical knowledge in the causal analysis of primary care data

Sara Geneletti[a*], Aidan G. O'Keeffe[b], Linda D. Sharples[c], Sylvia Richardson[d] and Gianluca Baio[b]

1. Simulation algorithm: Part I (simulating the treatment)

1. From the available subsample of THIN, we consider, for the i−th individual, the following observed variables: x_i^c as the centered version of the 10-year cardiovascular risk score; z_i as the threshold indicator, such that $z_i = 1$ if $x_i^c > 0$ (*i.e.* if the uncentered risk score $x_i > 0.2$) and $z_i = 0$ otherwise; t_i as the treatment indicator, where $t_i = 1$ if the individual receives statins and $t_i = 0$ otherwise; a_i as the observed age; d_i as an indicator of diabetes, taking value 1 if the i−th individual is diabetic and 0 otherwise; h_i as the individual HDL cholesterol level (measured in mmol/l); and y_i as the individual's LDL cholesterol level. These are the starting point for our simulations.
2. We then remove any pre-existing effects of treatment and threshold on the outcome y_i. This will allow the comparison of estimated results to a known, pre-specified, treatment effect (which we describe in Part II) as well as justifying the assumption that the outcome does not depend on the threshold. We fit the normal linear model:

$$y_i = \alpha_0 + \alpha_1 t_i + \alpha_2 z_i + \varepsilon_{1i} \tag{1}$$

with the ε_{1i} ($i \in \{1, \ldots, N\}$) independent, normal, zero mean error terms with a constant variance. We define $\boldsymbol{y}$ to be the vector of observed LDL cholesterol values, with sample mean $\bar{y}$, and $\hat{\boldsymbol{y}}$ as the vector of fitted values from the model (1). We draw a new set of simulated LDL cholesterol outcome values, denoted $\boldsymbol{y}^{\text{SIM1}}$ such that, for $i = 1, \ldots, N$:

$$y_i^{\text{SIM1}} = y_i - \hat{y}_i + w_i$$

with w_i drawn at random from a Normal$(\bar{y}, 0.1^2)$ distribution. We add the w_i term so that y_i^{SIM1} has approximately the same expectation as Y_i with the variance term included to reflect additional uncertainty in y_i^{SIM1} but still reasonably small because we do not expect y_i^{SIM1} to differ too greatly from y_i. A normal distribution is assumed for LDL cholesterol values, in general, throughout.
3. At this point, we define the individual probability of treatment $p_i = \Pr(T_i = 1)$ and fit the following generalised linear model:

$$\log\left(\frac{p_i}{1-p_i}\right) = \alpha_3 + \alpha_4 a_i + \alpha_5 d_i + \alpha_6 x_i^c + \alpha_7 h_i + \alpha_8 z_i.$$

[a] *Department of Statistics, London School of Economics*
[b] *Department of Statistical Science, University College London*
[c] *Leeds Institute of Clinical Trials Research, University of Leeds*
[d] *MRC Biostatistics Unit, Cambridge*
[†] Email: s.geneletti@lse.ac.uk

Copyright © 2013 John Wiley & Sons, Ltd.

Let $\hat{\boldsymbol{\alpha}}$ denote the estimates from the fitted model with corresponding estimated covariance matrix $\Sigma_{\boldsymbol{\alpha}}$. We re-draw the parameter estimates from a Normal$(\hat{\boldsymbol{\alpha}}, \Sigma_{\boldsymbol{\alpha}})$ distribution, which we indicate as $\tilde{\boldsymbol{\alpha}}$. This is usually done when imputing data to reflect our uncertainty in the estimated values of $\boldsymbol{\alpha}$. We replace $\tilde{\alpha}_7$ and $\tilde{\alpha}_8$ with pre-specified values to adjust the level of confounding and the strength of threshold as an instrument for treatment, respectively, in the simulated dataset.

4. For each individual, we estimate p_i (denoting the estimate $\hat{p}_i$) and randomly draw an estimated treatment variable, $\hat{t}_i$ from a Bernoulli$(\hat{p}_i)$ distribution.

2. Simulation algorithm: Part II (simulating the outcome)

1. From Part I, we form the following normal linear model for the adjusted LDL cholesterol values

$$y_i^{\text{SIM1}} = \gamma_0 + \gamma_1 \hat{t}_i + \varepsilon_{2i},$$

with the ε_{2i} $(i \in \{1, \ldots, N\})$ independent normal, zero mean, error terms with a constant variance. We fit this model to obtain maximum likelhood estimates of the paramters γ_0 and γ_1 and use these to obtain the vector of estimated residuals, $\hat{\boldsymbol{\varepsilon}}_2$.

2. We fit the normal linear model:

$$\hat{\varepsilon}_{2i} = \gamma_2 + \gamma_3 a_i + \gamma_4 d_i + \gamma_5 x_i^c + \varepsilon_{3i}$$

with the ε_{3i} $(i \in \{1, \ldots, N\})$ independent, normal, zero mean error terms with a constant variance. The maximum likelihood estimates of $(\gamma_2, \gamma_3, \gamma_4, \gamma_5)^{\mathsf{T}}$ are obtained and used to calculate the vector of fitted values from this model, We add to each fitted value its corresponding standard error estimate, denoting the resulting vector $\tilde{\boldsymbol{\varepsilon}}_2$. This is done to slightly perturb the fitted values of $\boldsymbol{\varepsilon}_2$, incorporating additional randomness and uncertainty into the estimated values. We then add this $\boldsymbol{\varepsilon}_2$ to $\boldsymbol{y}^{\text{SIM1}}$ to form a slightly distorted vector of simulated LDL cholesterol values, which we denote as $\boldsymbol{y}^{\text{SIM2}}$.

3. Finally, we add a treatment effect of a pre-specified size, τ by defining:

$$y_i^{\text{SIM3}} = y_i^{\text{SIM2}} + (1 - \hat{t}_i) v_{1i} + \hat{t}_i v_{2i},$$

where $v_{1i} \sim$ Normal$(0, 0.5^2)$ and $v_{2i} \sim$ Normal$(\tau, 0.5^2)$. The resulting vector $\boldsymbol{y}^{\text{SIM3}}$ is a set of simulated LDL cholesterol values with a treatment effect of size τ for the treated. A relatively small variance of 0.25 is chosen here so that the treatment effect can be distinguished.

2.1. *Unobserved Confounding*

The levels of unobserved confounding considered are defined using the correlation between the LDL and HDL cholesterol levels in the original data (we term this Corr(HDL,LDL)) and a pre-specified value of α_7 in Step 3 of Part I of the simulation algorithm. We describe these as follows:

- Level 1 - Corr$(HDL, LDL) = 0.18$, $\alpha_7 = 4$;
- Level 2 - Corr$(HDL, LDL) = 0.50$, $\alpha_7 = 4$;
- Level 3 - Corr$(HDL, LDL) = 0.18$, $\alpha_7 = -2$;
- Level 4 - Corr$(HDL, LDL) = 0.50$, $\alpha_7 = -2$.

3. Threshold as an Instrumental Variable

We pre-determine the value of α_8 in Step 3 of Part I of our simulation algorithm to adjust the level of unobserved confounding. We define:

$$\begin{aligned} \alpha_8 &= 10 \quad \text{where threshold is a strong instrument for treatment;} \\ \alpha_8 &= 4 \quad \text{where threshold is a weak instrument for treatment.} \end{aligned}$$

 Copyright © 2013 John Wiley & Sons, Ltd.

4. Plots for a weak instrument

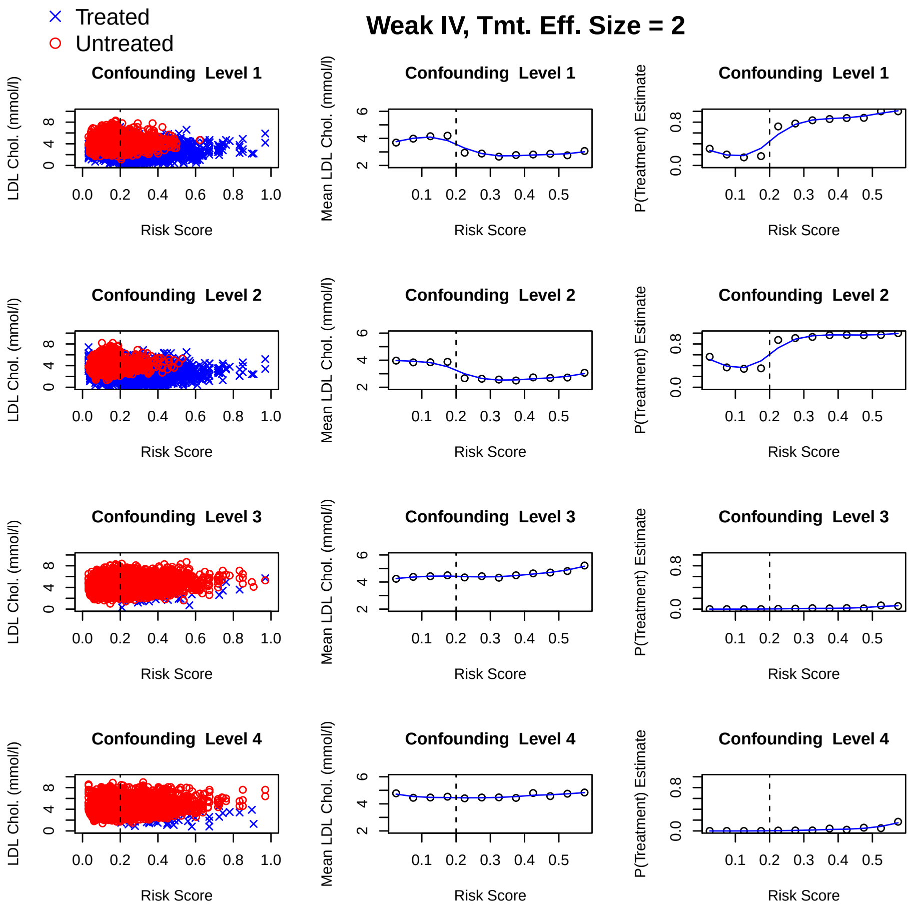

Figure 1. Plots in the left hand column show risk vs. simulated LDL cholesterol level, those in the central column show risk score (bin mid-point) vs. sample mean LDL cholesterol level and those in the right-hand column show risk score (bin-midpoint) vs. estimated probability of treatment. Plots are shown for different levels of confounding using simulated datasets with a treatment effect of size 2 and threshold acting as a weak instrument for treatment. A dashed vertical line indicates the threshold level.

Copyright © 2013 John Wiley & Sons, Ltd.
